# Cytomegalovirus infection and risk of preeclampsia: A meta-analysis of observational studies

**DOI:** 10.22088/cjim.9.3.211

**Published:** 2018

**Authors:** Zahra Geraili, Seyed Mohammad Riahi, Soghra Khani, Ali Rostami, Masomeh Bayani, Karimollah Hajian-Tilaki, Malihe Nourollahpour Shiadeh

**Affiliations:** 1 Department of Biostatistics and Epidemiology, School of Medicine, Babol University of Medical Sciences, Babol, Iran; 2 Faculty of Health, Birjand University of Medical Sciences, Birjand, Iran; 3 Department of Epidemiology, School of Public Health, Shahid Beheshti University of Medical Sciences, Tehran, Iran; 4 Sexual and Reproductive Health Research Center, Mazandaran University of Medical Sciences, Sari, Iran; 5 Department of Midwifery and Reproductive Health, Nursing and Midwifery School, Mazandaran University of Medical Sciences,Sari, Iran; 6 Infectious Diseases and Tropical Medicine Research Center, Health Research Institute, Babol University of Medical Sciences, Babol, Iran

**Keywords:** Cytomegalovirus infection, Pregnant women, Preeclampsia

## Abstract

**Background:**

Cytomegalovirus (CMV) infection is one of the most common infectious diseases in pregnant women in terms of global impact and is related with many adverse health consequences during pregnancy. For the first time, we performed a systematic review and meta-analysis study to evaluate the possible association between CMV infection and preeclampsia (PE).

**Methods:**

A comprehensive literature search **to **identify the relevant papers published earlier than February 2018 was performed in PubMed, ISI (Web of Science), Google Scholar and SCOPUS databases. We followed the PRISMA (preferred reporting items for systematic reviews and meta-analyses) guidelines for design, analysis and interpretation of results. Pooled odds ratio (OR) and 95% confidence intervals (CI) were estimated using a random-effects meta-analysis model. Heterogeneity was assessed with Q-test and *I*^2^ statistic**s**.

**Results:**

A total of 13 studies including 6158 pregnant women (2734 women with PE and 3424 healthy controls) met the eligibility criteria. The results of meta-analyses based on PCR (OR: 3.09; 95% CI:0.72–13.24; I^2^=57.3%), IgG-ELISA (OR: 1.24; 95% CI:0.83–1.85; I^2^=71%) and IgM-ELISA (OR: 1.04; 95% CI:0.66–1.65; I^2^=0.0%) demonstrated that CMV infection could not be a potential risk factor for PE.

**Conclusions:**

In conclusion, results of the present study demonstrated that CMV infection could not be a potential risk for developing PE. More epidemiological and experimental studies are needed to investigate the impact of CMV infection on the development of PE.

Preeclampsia (PE), a pregnancy-specific multisystem vascular disorder, is globally responsible for many maternal and perinatal deaths and complications, as it affects 2%- 8% of pregnancies (1). It generally occurs during the second half of pregnancy and characterized by new-onset gestational hypertension, generalized vascular dysfunction and proteinuria (2). Etiology of the disease is still unclear, although it is hypothesized that PE can be resulted from placental malperfusion due to abnormal remodeling of maternal spiral arteries, and both of maternal and fetal components are involved in the development of the condition (3). Moreover, it is demonstrated that PE is associated with disturbance in the levels of angiogenic factors including soluble fms-like tyrosine kinase 1 (sFlt-1) and placental growth factor (PlGF) (4, 5). Furthermore, some studies showed that PE is related with increased levels of maternal circulating cytokines such as TNF- α, IFN- γ, IL- 10 and IL- 6 (6, 7). In the last two decades, there is growing evidence that bacterial, viral and parasitic infectious agents and their related inflammatory responses are implicated in the pathophysiology of PE (1, 8, 9). Cytomegalovirus (CMV), a ubiquitous beta herpes virus, is globally one of the most common congenital infectious agents during pregnancy.

CMV is responsible for a broad range of disabilities in newborns, such as visual impairment, sensorineural hearing loss, and motor and cognitive deficits. In pregnant women, clinical symptoms of CMV infection is generally nonspecific and resemble a mononucleosis- or flu-like syndrome, with fever, sore throat, cervical lymphadenopathy, myalgia, and fatigue (10, 11). 

Moreover, it is demonstrated that CMV infection is a potential risk factor for the development of hypertension and cardiovascular disease (12). In the past two decades, several observational and experimental studies have investigated the possible relationship between CMV infection and the development of PE in pregnant women. However, the majority of studies demonstrated conflicting results together and to date, it remains unknown whether women with CMV infection are at increased of developing preeclampsia or not; therefore, for the first time we designed this systematic review and meta-analysis study in an attempt to a better understanding of a possible link between CMV infection and development of PE.

## Methods

**Study design: **For this meta-analysis study, we followed the PRISMA (preferred reporting items for systematic reviews and meta-analysis) guidelines for design, analysis and interpretation of results. The exposure for our study was serological or molecular evidence of CMV infection and the main outcome of interest was developing PE in infected women and compared with healthy controls. To prevent common biases during the literature research, language, geographical area or date restrictions were not applied. All observational (cross-sectional, cohort, or case–control) studies that evaluated the CMV infection status among women with PE and healthy controls were included in the present meta- analysis. 

PE is defined as a systolic blood pressure ≥ 140 mm by the American College of Obstetricians and Gynecologists (ACOG) criteria (13) and CMV infection is measured by the detection of serum CMV-specific IgG or IgM using serological methods and DNA-based detection molecular methods. We excluded case- reports, experimental studies and non-original papers (review, systematic reviews, editorials, or letters).

**Figure 1:**
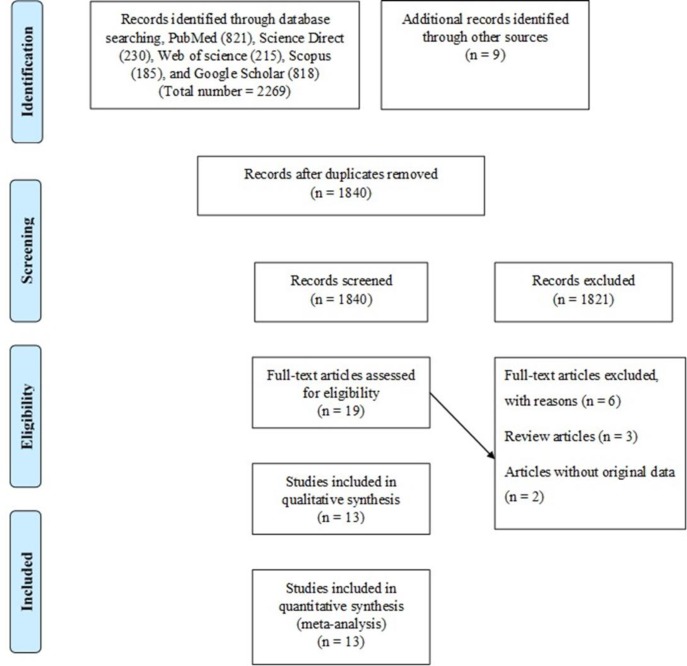
Flow chart showing the process of study selection

**Search strategy and study selection: **A comprehensive literature search for identifying the relevant papers published earlier than February 2018 was performed in PubMed, ISI (Web of Science), Google Scholar and SCOPUS databases. Our search was done by using the combination of the following keywords: “Cytomegalovirus”, “Cytomegalovirus infection”, “CMV”, “preeclampsia”, “eclampsia”, “gestosis”, “pregnancy hypertension” and “gestational hypertension”, with the Boolean operators “OR” and/or “AND”. Finally, our search was complemented by reviewing the reference lists of the electronically retrieved full-text papers and related reviews. Our search strategy is schematically presented in the PRISMA flow diagram (figure 1 and supplementary figure 1).

**Data extraction and study quality assessment: **After duplicate removal, two researchers (Z.G. and A.R) independently assessed the full text of eligible studies and information was extracted using Microsoft Excel software. The following information was extracted for each study: first author, publication year, country, study design, sample size, diagnostic method. In case-control studies, matching criteria, number of cases and controls and of infected subjects in each group were extracted. 

Any uncertainties were resolved by joint review of the manuscript and consultation with third researcher (M.N.S) to reach consensus. Newcastle-Ottawa scale was used for quality assessment of the included studies, as it was used in our previously meta-analysis studies (14, 8). This step was also done by two researchers (Z.G. and A.R) and disagreements were resolved after consultation with third researcher (M.N.S).

**Data synthesis and statistical analysis: **In this study, meta-analysis was performed using Stata Version 12 (Stata Corp., College Station, TX). The association between CMV infection and PE was assessed through generating the pooled odds ratio (OR) and 95% confidence intervals (CI) using a random effects model. 

In studies where adjusted ORs were presented, they were used in preference to crude odds ratio. To assess heterogeneity amongst the studies, Q-test and *I*^2^ methods were utilized. Stratified analysis was subsequently performed regarding the type of antibodies and methods (IgG, IgM and PCR). Publication bias was assessed through Egger’s publication bias method for asymmetry. 

A p*-*value of less than 0.1 was considered statistically significant. Results are presented as forest plots and the association between CMV infection and PE is illustrated by an OR and 95% CI.

## Results

**Study characteristics: **As shown in PRISMA flowchart (figure 1), our literature search yielded 2278 articles. After duplicate removal and assessment of the titles and abstracts, 19 studies were reviewed in full text. Of these, six studies were excluded after applying inclusion and exclusion criteria, and finally 13 articles met the eligibility criteria to be included in the meta-analysis (15-27). Among 13 included studies, eight had a case-control design and five were nested case-control studies. The eligible studies were performed in seven different countries from three continents (nine in America, three in Europe and one in Asia). Two studies were based on molecular methods, 11 studies used serological methods to detect CMV infection in cases and controls. Four studies included reported seroprevalence of CMV infection based on the detection of only IgG antibodies, three on IgM antibodies, and four studies screened both the IgG and IgM antibodies for CMV infection. A total of 2734 pregnant women with confirmed PE were included in these studies. All studies used ACOG criteria for the selection of preeclamptic women. As well, the included studies recruited a total of 3424 healthy pregnant women as controls. The main characteristics for included studies are presented in table 1.

**Prevalence of CMV infection among preeclamptic women and controls: **Overall, the prevalence of CMV infection in pregnant women with PE based on PCR, IgG and IgM were 40.6% (24/59), 58.8 (1123/1909), and 3.5% (89/2490), respectively. While, the prevalence of CMV infection in healthy controls based on PCR, IgG and IgM were 21.5% (14/65), 62.1% (1595/2566), and 3.5% (74/2099), respectively.

**Table 1:** Summary of studies investigating the association between Cytomegalovirus infection and preeclampsia

**First author/year**	**Ref**	**Country**	**Diagnostic Method**	**Cases**		**Controls**		**Quality score**
				**Number**	**Infected**	**Number**	**Infected**	
Belfort, 1998		USA	PCR	33	10	36	2	5
Trogstad, 2001		Noewey	IgG-ELISA	33	19	945	650	7
Carreiras, 2002		Venezuela	PCR	26	14	29	12	6
Dadelszen, 2003		Canada	IgG-ELISA	38	30	113	92	6
Rustvuld, 2005		USA	IgG-ELISA	48	21	140	60	7
Xie, 2010		USA	IgG&IgM-ELISA	78	IgG, 41; IgM, 8	109	IgG, 29; IgM, 7	7
Strand, 2012		Norway	IgG&IgM-ELISA	1470	IgG, 819; IgM, 18	991	IgG, 578; IgM, 12	7
Zhang, 2012		China	IgM-ELISA	52	49	34	34	5
Haggerty, 2013		USA	IgM-ELISA	509	7	336	4	7
Haggerty, 2013		USA	IgM-ELISA	205	4	423	13	5
Xie, 2014		USA	IgG&IgM-ELISA	30	IgG, 17; IgM, 3	60	IgG, 22; IgM, 3	5
Radulescu, 2016		Romania	IgG-ELISA	66	40	62	26	6
Alvarado-Esquivel, 2017		Mexico	IgG&IgM-ELISA	146	IgG, 136; IgM, 0	146	IgG, 138; IgM, 1	6

**Figure 2:**
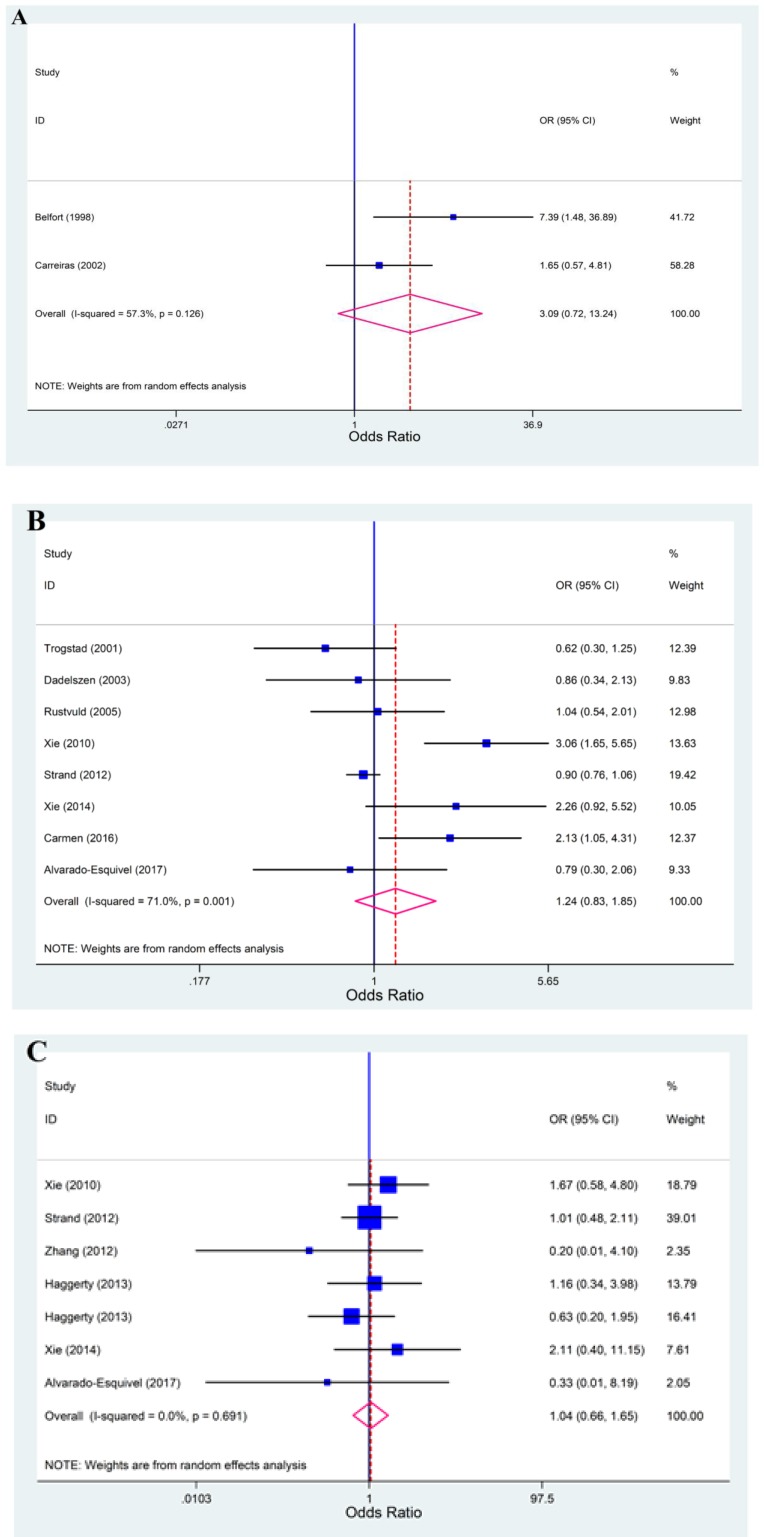
Forest plots for the association between cytomegalovirus infection and preeclampsia. A) Forest plot regarding the PCR method, B) Forest plot regarding the IgG-ELISA method, C) Forest plot regarding the IgM-ELISA method

**Results of meta-analysis: **At first, we performed meta-analysis regarding the three different methods (PCR, IgG-ELISA and IgM-ELISA) used in the included studies for the detection of CMV infection between cases and controls. The results of meta-analysis based on PCR (OR: 3.09; 95% CI:0.72–13.24; I^2^=57.3%), IgG-ELISA (OR: 1.24; 95% CI:0.83–1.85; I^2^=71%) and IgM-ELISA (OR: 1.04; 95% CI:0.66–1.65; I^2^=0.0%) demonstrated that CMV infection could not be the potential risk factor for PE (figure 2, A-C). 

**Figure 3:**
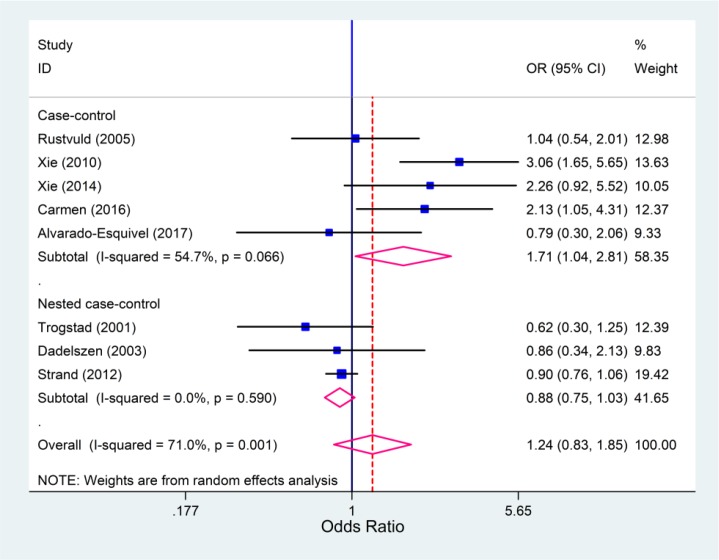
Forest plots for the association between cytomegalovirus infection and preeclampsia, result of the subgroup analysis

**Figure 4:**
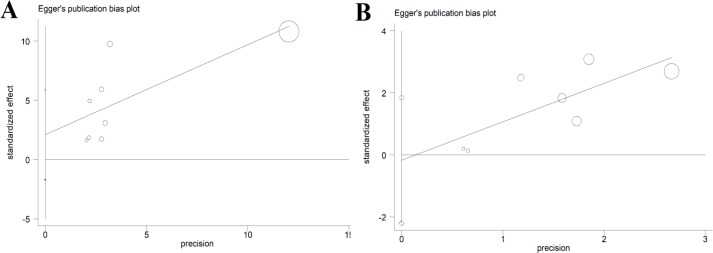
Publication bias using Egger’s plot. (A) Publication bias related with IgG-ELISA method. (B) Publication bias related with IgM-ELISA method.

In subgroup analysis regarding the IgG antibody and type of studies, a significant relationship was observed in case-control studies (OR: 1.71; 95% CI:1.04–2.81; I^2^=54.7%), whereas this relationship was not significant in nested case-control studies (OR: 0.88; 95% CI:0.75–1.03; I^2^=0.0%) (figure 3). Egger’s test was used to detect the possibility of publication bias. Based on the symmetry assumption, there was no significant publication bias in the studies (figure 4 A and B).

## Discussion

The results of this study demonstrated that women with PE have higher rate of CMV infection compared to healthy controls, although the statistically significant relationship was not observed. Specially we have found higher odds in studies that used PCR to detect CMV infection (OR=3.09), although the number of studies was very low (two studies). We guess if more molecular studies were available, it was possible that significant correlation would be found. Higher odds found in PCR studies could be due to higher sensitivity and specificity of PCR-based methods compared to serological methods.

Although it should be more investigated, since several potential mechanisms could be involved in the association between CMV infection and the development of PE. Endothelial-platelet dysfunction and acute atherosis in arterial wall resulting from CMV infection can lead to uteroplacental ischemia, a key pathogenic condition to the development of PE (28, 29). Further, CMV infection could stimulate the release of immune inflammatory mediators including, tumour necrosis factor alpha (TNF-α), interferon gamma (IFN-γ), interleukines (IL-4, IL-8, IL-18), neutrophils and is related with upregulated expression of TLR-2, and TLR-4 (26, 30-32). 

Several studies have demonstrated that the shift of immunological cytokine profile of Th2 toward Th1 and high levels of pro-inflammatory cytokines have important role in the onset of PE (1, 33, 34). Moreover, increasing evidence has shown that the extent of neutrophil activation and upregulated TLR expression can be linked with the pathogenesis of PE (32, 35). 

In addition, it is demonstrated that CMV infection can modify adhesion molecules such as VCAM-1 (vascular cell adhesion molecule-1) and ICAM-1 (intercellular adhesion molecule-1) and E-selectin which is related with the increase of matrix metalloproteinase (MMP-2) activity and fms-like tyrosine kinase-1 (sFlt-1), that can cause alteration in vessel remodeling, infiltration of inflammatory cells and the development of PE (36, 26).

The strength of our study includes rigorous methodology, the use of ACOG criteria to identify the cases in all studies, well-defined matching criteria for controls, and subgroup analysis considering the type of studies and diagnostic methods. Yet like our previous meta-analysis studies (14, 8), this study was limited by the fact that, low number studies with mostly low sample size were available in this issue. Additionally, most of the included studies have a case-control design, while other type of observational studies like cohorts could be more useful to better understand possible relationship. Another limitation is the fact that the results of the present meta-analysis are based on the data from studies with different methodological characteristics, and different cut-offs in serological methods, however we used random effects to overcome these limitations. Moreover, like our previous study on *Helicobacter pylori* and the developing of PE (8), the present study was also copied with regard to the results without adjustment for potential confounders in some included studies and restricted geographical areas (America, Europe and one study in Asia). Furthermore, since CMV infection is ubiquitous and determining IgG is not a reliable method for CMV primary infection, therefore, it can be a source of heterogeneity in the current study.

In conclusion, the findings of the present study demonstrated that CMV infection could be a potential risk for developing PE. More epidemiological studies (especially longitudinal studies using both molecular and serological methods) are needed to investigate the impact of CMV infection or its eradication on the risk of developing PE. Besides, more experimental studies should be done to identify the exact involved pathways. The GRADE for this study is this fact that further researches are very likely to have an important impact on our estimated effect and is likely to change the estimate. 
